# Supplementary material for: Loss of the PPE71-esxX-esxY-PPE38 locus drives adaptive transcriptional responses and hypervirulence of Mycobacterium tuberculosis lineage 2
Source: Sci Adv. 2025 Jul 2;11(27):eadw5194. doi: 10.1126/sciadv.adw5194 (PMC12219505; doi:10.1126/sciadv.adw5194)
Supplement: Supplementary file 1 — Figs. S1 to S6 Legends for tables S1 to S3 Tables S4 and S5 [file sciadv.adw5194_sm.pdf]

Science Advances
AAAS

Supplementary Materials for

Loss of the *PPE71-esxX-esxY-PPE38* locus drives adaptive transcriptional responses and hypervirulence of *Mycobacterium tuberculosis* lineage 2

Benjamin Koleske *et al.*

Corresponding author: William R. Bishai, wbishai1@jhmi.edu

Sci. Adv. **11**, eadw5194 (2025)
DOI: 10.1126/sciadv.adw5194

The PDF file includes:

Figs. S1 to S6
Legends for tables S1 to S3
Tables S4 and S5

Other Supplementary Material for this manuscript includes the following:

Tables S1 to S3

Supplementary Figures

A

Strain	Day 1 log_{10}(CFU)
WT+vector	2.55
WT+71OE	2.39
Δ7138+vector	2.65
Δ7138+71	2.75
Δ7138+71XY	2.61
Δ7138+71XY38	2.80
Δ7138+71OE	2.78

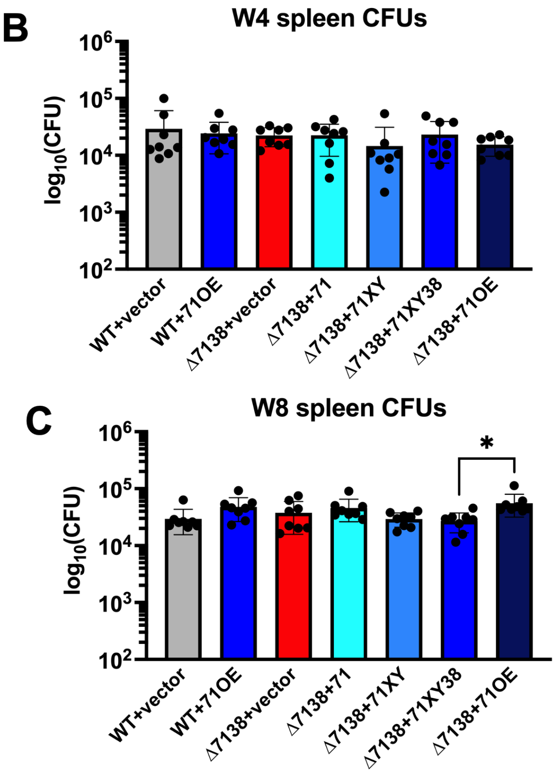

Figure S1: H37Rv *PPE71* variant strains showed equivalent inocula and minimal spleen findings.
(A) Day 1 lung CFUs to assess lung inocula for BALB/c infection with *M.tb* H37Rv *PPE71* variant strains. No strains had significantly different inocula from the WT+vector strain by one-way ANOVA. (n=3-4.)
(B–C) Spleen CFUs from Week 4 (E) and Week 8 (F) timepoints. All significant comparisons by one-way ANOVA are depicted. (mean±SD; n=8 each, *: $p<0.05$.)

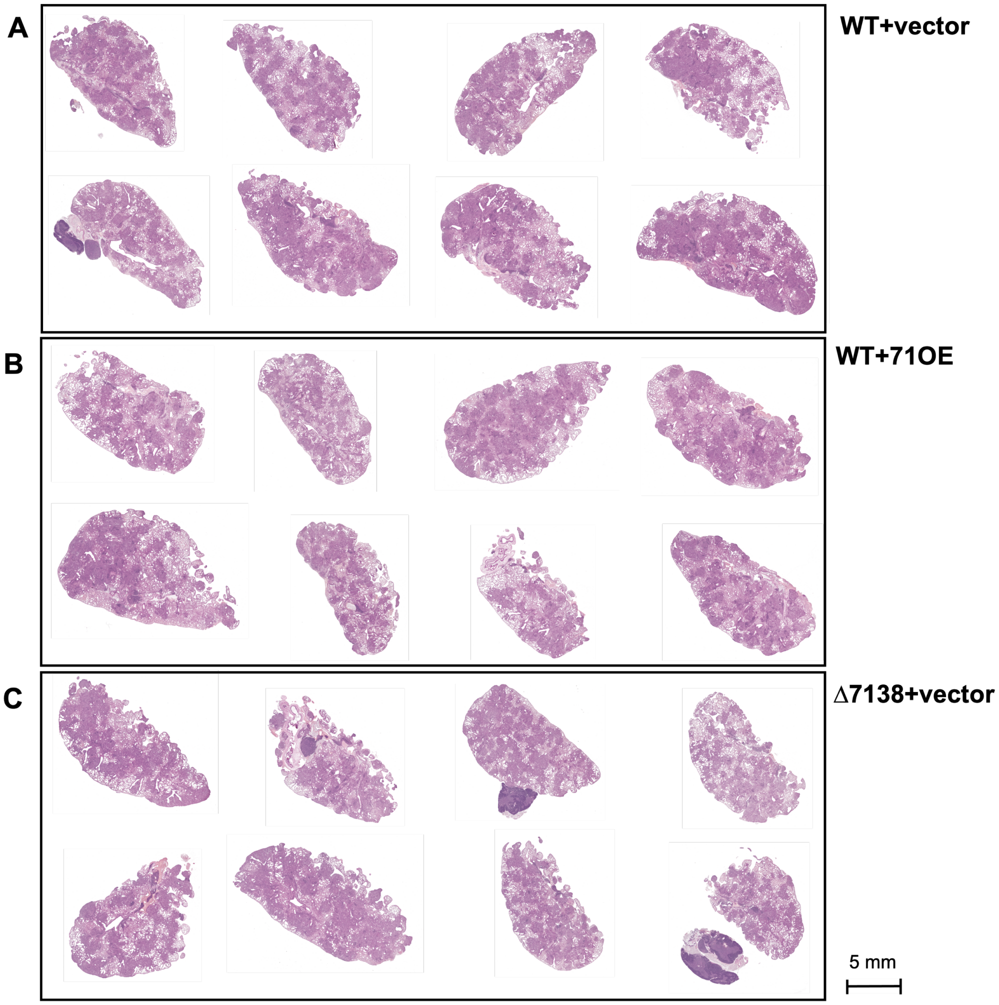

Figure S2: Complete set of Week 8 lung histology images for WT+vector, WT+71OE, and Δ7138+vector mouse groups.

(A–C) H&E histology images of lungs taken from female BALB/c mice infected with WT+vector (A), WT+71OE (B), or Δ7138+vector (C) strains at the Week 8 timepoint. These images were used to quantify the percent area of each lung occupied by inflammatory lesions. Non-pulmonary tissue present in the images (chiefly, upper airway and mediastinal lymph nodes) were excluded from this analysis.

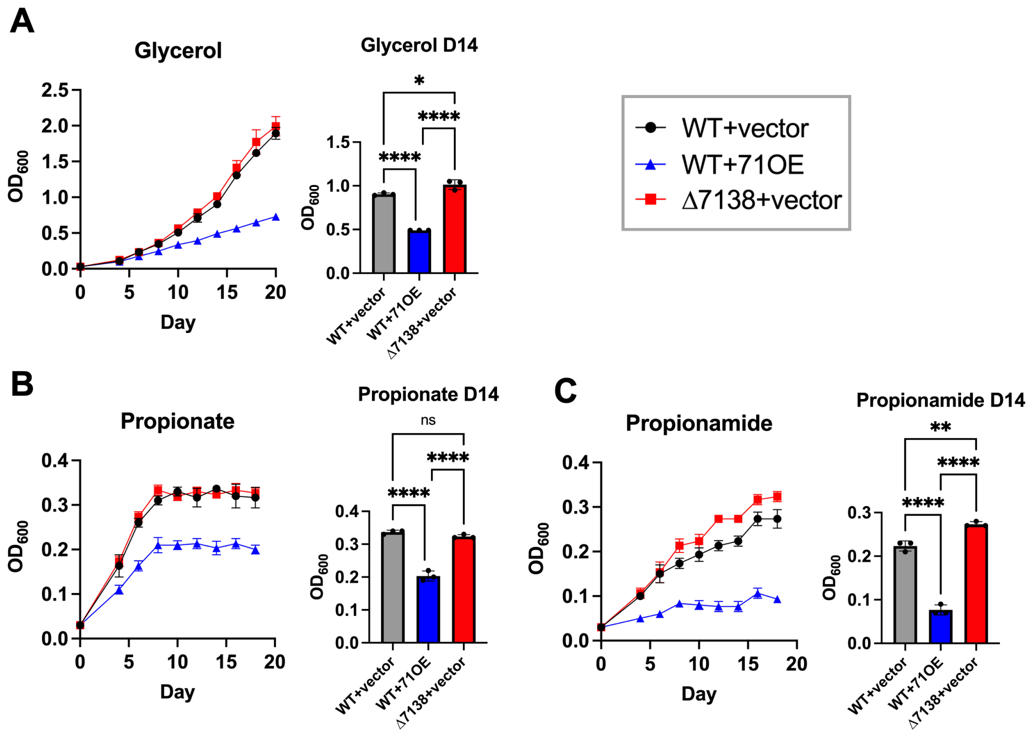

Figure S3: H37Rv WT+71OE has a growth defect exacerbated by particular carbon sources.

(A–C) Growth curves of *M.tb* strains in minimal 7H9 broth supplemented with 0.05% tyloxapol and each of (A) 0.2% glycerol, (B) 0.1% propionate, or (C) 5 mM propionamide. Graphs of optical densities at 600 nm (OD_{600}) for the Day 14 timepoints are provided for each medium. (mean±SD; n=3, ns: non-significant; *: p<0.05; **: p<0.01; ****: p<0.0001 by one-way ANOVA.)

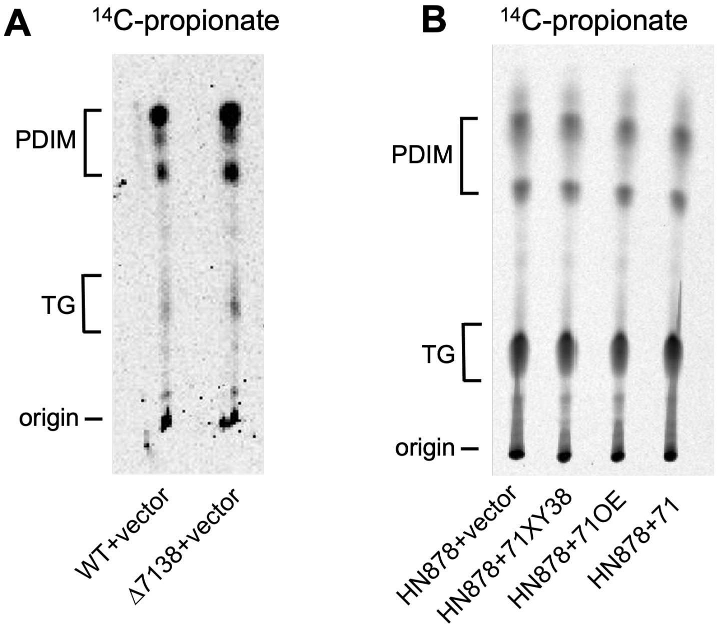

Figure S4: M.tb H37Rv Δ7138 overproduces triglycerides.
(A–B) TLC plates with apolar lipid fractions extracted from H37Rv WT+vector and Δ7138+vector strains (A) or HN878+vector, HN878+71XY38, HN878+71OE, and HN878+71 strains (B) labeled with ^{14}C-propionate and visualized by phosphorimaging. Positions of phthiocerol dimycocerosate (PDIM) and triglyceride (TG) lipids, as well as the origin, are indicated.

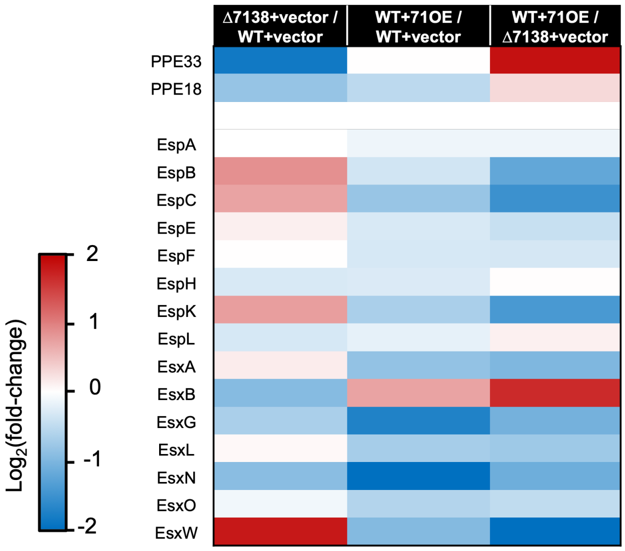

Figure S5: Type VII secretion system substrates detected in culture filtrate.
Heat map of $\log_2$ fold-changes between each pair of strains for PE/PPE-, Esp-, and Esx- family proteins detected in CF fractions by mass spectrometry.

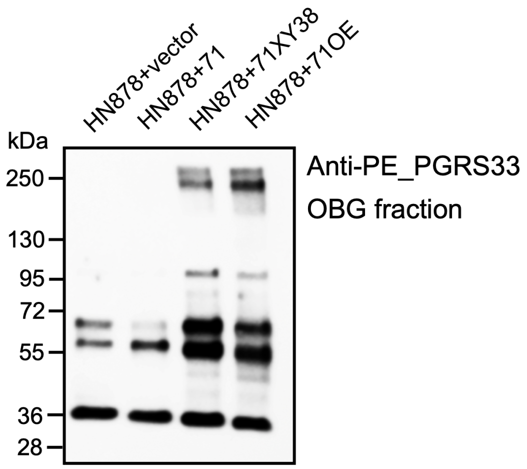

Figure S6: Restoring the *PPE71* locus into HN878 strains boosts PE_PGRS protein secretion.

HN878+vector, HN878+71, HN878+71XY38, and HN878+71OE cultures were incubated overnight in complete 7H9 broth without detergent. The octyl glucoside (OBG) fraction was subjected to Western blotting with anti-PE_PGRS33 antibody. Additional bands seen in the HN878+71XY38 and HN878+71OE strains suggest the presence of a broader range of PE_PGRS proteins. (kDa: kilodaltons.)

Supplementary Tables

Table S1. RNAseq output for transcripts from WT+vector, WT+71OE, and Δ7138+vector strains.
Raw counts and normalized counts (scaled by total transcripts per sample) are provided. Fold-changes and adjusted p-values (using a Benjamini-Hochberg multiple test correction) were computed for each comparison.

Table S2. Mass spectrometry output for the culture filtrate fraction of WT+vector, WT+71OE, and Δ7138+vector strains.
Raw input and quantified proteins, normalized to total peptides per sample by summed signal/noise (S/N) ratio, are provided. Fold-changes and p-values were computed for each comparison.

Table S3. Mass spectrometry output for the octyl glucoside fraction of WT+vector, WT+71OE, and Δ7138+vector strains.
Raw input and quantified proteins, normalized to total peptides per sample by summed signal/noise (S/N) ratio, are provided. Fold-changes and p-values were computed for each comparison.

**Table S4. Plasmids used in this study.**		
**Plasmid**	**Description**	**Reference**
pMH94	L5 *int*, *M.smegmatis attP*, *ampR* (*E.coli*), *kanR* (*M.tb*)	(71)
pMH94-PPE71	pMH94 + [422nt upstream of *PPE71* to 111nt downstream of *PPE71*]	This work
pMH94-71XY	pMH94 + [414nt upstream of *PPE71* to 177nt downstream of *esxY*]	This work
pMH94-71XY38	pMH94 + [414nt upstream of *PPE71* to 244nt downstream of *PPE38*]	This work
pSD5	*OriM*, *M.leprae hsp65* promoter, *ampR* (*E.coli*), *kanR* (*M.tb*)	(72)
pSD5-71OE	pSD5 + [60nt upstream of *PPE71* to 28nt downstream of *PPE38*]	This work
pET-28a(+)	T7 promoter, lac operator, 6xHis-insert-6xHis, *kanR* (*E.coli*)	Novagen
pET28a-PPE71	pET-28a(+) + *PPE71* [in frame with both N-terminal and C-terminal 6xHis]	This work

Table S5. Oligonucleotides used in this study.	
**Oligonucleotide**	**Sequence (5'–3')**
PPE71_24f	GAATTCGAGCTCGGTACCCGGGGATCCTCTAGATTCCGTTCGGTAGTGCGAT
PPE71_24r	GCAGAGATGGTGCCCTTGGTGGTCGACTCTAGTCATAAACCGAGTAGCCACCA
PPE71locus_59_F	GACCTCGGTACCAGGTAGTGCGATGTAGTTGGTCT
EsxY_59_R	GTCGACTCTAGACTCAACGGCTCAGACACAAAC
PPE38term_59_R	GTCGACTCTAGATCCTTCAGATCGCGATGGTTG
PPE71_pSD5_f	GCGATATCCGGAGGAATCACTTCCATGTTTGTGTCTGGAGAGTGGTAGG
PPE71_pSD5_r	CCATTGAAGACCGGGCCAGAACGCGGCAAAGACCCCGACCAATC
dPPE38_57_f	GTCACTAGACAGTTCGAGGTCA
dPPE38_57_r	GGTAGTGCGATGTAGTTGGTCT
PPE71_34F	ACCGAAAACCTTTACTTCCAGGGCCATATGTGGAGAGTGGTAGGCGGA
PPE71_34R	TGCTCGAGTGCGGCCGCAAGCTTGTCGACCCAATCACCTCCGCCGTATCC
pMH94_Fseq	GAAAATACCGCATCAGG
pMH94_Rseq	GAATAGACCGGGACAAGG
PPE71-EsxX_Fseq	GTCTTTGCGTTGATGACAT
PPE71-EsxX_Rseq	ATGTCATCAACGCAAAGAC
EsxY-PPE38_Fseq	CCTTCGGCATGTCAACA
EsxY-PPE38_Rseq	TGTTGACATGCCGAAGG
pSD5_insert_FWD	AGCGTAAGTAATGGGGGTTGTCG
pSD5_insert_REV	ATATATTCGGTCGCTGAGGCTTG
dPPE38_inner_f	CAGCGTCCGTACCATG
dPPE38_inner_r	TTCGCGCAGTCTTTACG
T7_FWDseq	TAATACGACTCACTATAGGG
T7_REVseq	GCTAGTTATTGCTCAGCGG
16S_q_F	GCCGTAAACGGTGGGTACTA
16S_q_R	TGCATGTCAAACCCAGGTAA
PPE71_q_T1F	ATCACCGCATCAAACGAGGA
PPE71_q_T1R	TGGATTTTTCGTGGTTGCCG
EccC5_qF	AGGCTTGGTTCTTCGCTCTC
EccC5_qR	AGATGTCTCGGGCACCAATG
pfkB_q_1F	TTTCCCAAGCGAACGGACTT
pfkB_q_1R	GCGAGCCATCGATTTTCGTC
devS_q_9F	CCGAGTGATACCCTGCGATG
devS_q_9R	CCGCACGAACGTAATCCTGA
devR_q_5F	CGCCAACTCCATTCCCTTGA
devR_q_5R	GCTGTCTGATCCTCACGTCC
PPE51_q_2F	CGCAGGATGTCGAGTCCTTT
PPE51_q_2R	AACTCGCCGATCCCAAAGTC
radA_q_1F	GTCGACGTTGTGCTGCATTT
radA_q_1R	GTGCAGGAGGAAACATCCGA
hspX_q_1F	TGGACCGGATCTGAATGTGC
hspX_q_1R	CCACCTACGACAAGGGCATT
narX_q_4F	ATAGTCGGTCTCCTGCGTCT
narX_q_4R	TCCACACACGGGGTGAATTG
kasA_q_6F	ATGCTCATCGAGACGGAGGA
kasA_q_6R	ATGAAAGGCGTCCGAGGTG
fas_q_1F	GCCCCATCATCTGGAAGACC
fas_q_1R	CATCTCCAAGCACGACACCT
tgs1_q_3F	GCAGGTTGGGCAGCATTAAC
tgs1_q_3R	TCAATGATGTTGCGCTTGCC
